# Feasibility and behavioral effects of prolonged static and dynamic standing as compared to sitting in older adults with type 2 diabetes mellitus

**DOI:** 10.1186/s12877-020-01600-0

**Published:** 2020-06-11

**Authors:** Uros Marusic, Martijn L. T. M. Müller, Neil B. Alexander, Nicolaas I. Bohnen

**Affiliations:** 1 Functional Neuroimaging, Cognitive and Mobility Laboratory, Department of Radiology, University of Michigan, 24 Frank Lloyd Wright Drive, Domino’s farms, Lobby B-1000, Ann Arbor, MI 48106 USA; 2 Institute for Kinesiology Research, Science and Research Centre Koper, Koper, Slovenia EU; 3 Department of Health Sciences, Alma Mater Europaea – ECM, Maribor, Slovenia EU; 4 Morris K. Udall Center of Excellence for Parkinson’s Disease Research, University of Michigan, Ann Arbor, Michigan USA; 5 Division of Geriatric and Palliative Medicine, Department of Internal Medicine, University of Michigan, Ann Arbor, Michigan USA; 6 Geriatric Research Education and Clinical Center, Veterans Affairs Ann Arbor Healthcare System, Ann Arbor, Michigan USA; 7 Department of Neurology, University of Michigan, Ann Arbor, Michigan USA

**Keywords:** Non-exercise physical activity, Cognitive functioning, Executive functions, Metabolic consumption, Ergonomic adjustment, Active working place

## Abstract

**Background:**

Physical inactivity is prevalent in older adults with type 2 diabetes mellitus (T2DM) and may exacerbate their clinical symptoms. The aim of this study was to examine the feasibility of 4-h regular versus more dynamic standing sessions while performing routine desktop activities as a non-exercise physical activity intervention in older adults with T2DM to increase non-exercise activity.

**Methods:**

Twelve older adult patients with T2DM (3 female; age 71 ± 4 years; Body mass index 34 ± 5 kg/m^2^) completed three sessions (baseline sitting followed by “static” or “dynamic” desktop standing sessions). Participants stood behind a regular height-adjustable desk in the “static” standing session. An upright dynamic standing desk, which provides cues to make small weight-shifting movements, was used for the “dynamic” standing session. Oxygen consumption, cognitive performance, as well as net standing duration, total movement activity, and musculoskeletal discomfort were assessed during all three sessions.

**Results:**

All participants were able to complete all sessions. Oxygen consumption and overall movements progressively increased from sitting to static and dynamic standing, respectively (*p* < 0.001). The duration of breaks during standing (*p* = 0.024) and rate of total musculoskeletal discomfort development (*p* = 0.043) were lower in the dynamic standing compared to static standing sessions. There was no evidence of executive cognitive worsening during either standing session compared to sitting.

**Conclusions:**

Prolonged 4-h standing as a simple non-exercise physical intervention is feasible in older adults with T2DM and may have metabolic (oxygen consumption) benefits. Increasing movement during desktop standing may offer incremental benefits compared to regular standing. Prolonged desktop standing might provide an effective intervention in T2DM older participants to target sedentariness.

**Trial registration:**

ClinicalTrials.gov (NCT04410055), retrospectively registered May 27, 2020.

## Background

Diabetes mellitus is highly prevalent in older adults [1] and is expected to reach endemic proportions as it is predicted to increase by 54% from 2015 to 2030 in the United States of America [2]. Over 30% of older adults probably have evidence of pre-diabetic insulin resistance [3]. Type 2 diabetes mellitus (T2DM) in older adults is especially problematic as it impacts both physical and cognitive function [4–6]. Hence, effective control of T2DM and its sequelae is of utmost importance in this geriatric population.

Sedentary behavior has been strongly associated with the development of diabetes mellitus [7]. Unfortunately, the modern home- and workplace environment promote sedentary behavior. This is further exacerbated by significant barriers to exercise and physical activity in T2DM patients, such as poor tolerance to physical effort, development of pain with physical activity, and lack of motivation [8]. Older adult T2DM patients are more likely to be physically inactive and adherence to an exercise program to meet recommended minimum guidelines appears to be an elusive goal in these patients [9].

Simple intermittent non-exercise interruption of prolonged inactivity during daily life could potentially represent a more feasible target in older adult T2DM patients [10]. For example, even a modest increase in physical activity and reduction in sedentary time may significantly improve health, especially in the most inactive older adults [11]. Upright standing represents the most rudimentary form of physical activity and may have specific metabolic benefits in T2DM. Several studies, mainly conducted in office workers, have shown beneficial effects of desktop standing on metabolic risk factors such as insulin resistance and glycemic excursion [12–14]. Non-exercise physical activity interventions that can be performed at home while integrating this with routine activities of daily living may provide an attractive means to reduce sedentariness and improve diabetic control in older adult patients with diabetes.

The aim of this study was to examine the feasibility of 4h standing sessions while performing routine desktop activities as a non-exercise physical activity intervention in older adults with T2DM to increase non-exercise activity thermogenesis (NEAT). The primary hypothesis was that 4h standing behind a height-adjustable desk is feasible in an older adult population with T2DM. We also hypothesized that standing while performing weight-shifting movements will lead to additional increase in oxygen uptake as compared to normal more ‘static’ stance. Finally, we explored secondary outcome parameters of net duration of standing time, total movement, rate of musculoskeletal discomfort development and leg swelling, and performance on a cognitive task during the different sessions.

## Methods

### Study design and participants

Randomized cross-over study design was used to compare two different types of standing interventions. Twelve obese and overweight (Body mass index above 25 and less than 40 kg/m^2^) older participants (age range = 65–78 years) with clinically confirmed T2DM were recruited from flyers available to previous University of Michigan T2DM research participants. Four participants reported their daily sitting rates as less than 6 h/day, while eight rated their daily sitting duration between 6 and 10 h/day. Participants’ demographic and clinical characteristics are presented in Table 1. All subjects completed a 3-h sitting session prior to randomization to the cross-over study. All participants underwent a short clinical and motor function test battery pre- and post-study.

**Table 1:** Participants’ demographic and clinical characteristics with averages ± standard deviations

Age (years)	71.1 ± 3.8
Gender (M/F)	9/3
Height (m)	1.74 ± 0.09
Body Weight (kg)	101.5 ± 17.4
BMI (kg/m^2^)	33.5 ± 4.9

Note: *BMI* body mass index

#### Ethics

This study was approved by The University of Michigan Institutional Review Board. The study was conducted in accordance with the Declaration of Helsinki and all participants provided written informed consent.

### Study protocol

Participants stood behind a height adjustable table that was positioned about 0.5 m from a wall and ergonomically adjusted to each individual, in agreement with the United States Department of Labor - Occupational Safety and Health Administration (OSHA) recommendations (OSHA, n.d.-a). For the “static standing” position participants were asked to stand behind the table and there were *no* specific restrictions imposed for standing (e.g. “do not move” or “stand quietly”). This allowed participants to develop an individualized standing pattern. For the “dynamic standing” condition participants stood behind the same table but received periodic cues to induce weight-shifting steps. In both conditions, participants were allowed to shift their weight as often as desired with instructions to stand close to the tabletop. Leaning on the tabletop was permitted. Each of the two standing sessions did not exceed 4 h. Participants stood on an anti-fatigue mat during each of the standing sessions.

A seated control session condition was also used to get baseline measures for the primary variable (resting state oxygen consumption; VO_2_). The seated session did not exceed 3 h and participants remained seated throughout the test period except for the rare break.

The experimental testing protocol was conducted over three separate days, with each test day dedicated to one of the testing conditions. The sitting (baseline) condition was conducted first by all participants, followed by either static or dynamic condition (randomly assigned). For each of the conditions as many rest breaks were provided to the participants as needed. The same test battery was administered during each of the conditions.

### Instrumentation

#### Descriptive measures of user behavior and experience of table use

##### Number and duration of breaks taken during sitting and standing conditions

Participants could alternate between standing and sitting down ad libitum. Total time spent standing and sitting and the number, nature, and duration of breaks were recorded.

##### Total movements

During all three conditions participants wore a tri-axial accelerometer (Actigraph GT3X+, Actigraph Inc., Florida, USA), which was placed at the right side of the hip. Participants’ activity was recorded at 30 Hz and aggregated to 10-s epochs. Raw accelerometer activity counts were first manually cleaned, eliminating time periods spent on breaks, and further processed in MATLAB®. Overall movement represented in counts per minute were calculated as magnitude vector [15].

##### Musculoskeletal discomfort rates

Participants were asked to report musculoskeletal discomfort at the beginning and then every 30 min using a 100-mm visual analogue scale. Specifically, the participants indicated the location of the discomfort by marking an ‘X’ on a drawing of a human body, as well as its severity by drawing a vertical line along a horizontal line beginning at “No discomfort at all” and ending at “Worst discomfort ever experienced” [16]. Discomfort was quantified on this visual analogue scale by measuring the horizontal distance between the first vertical line (i.e., “No discomfort at all”) and the line drawn by the participants, with potential ratings ranging from a minimum of 0 mm to a maximum of 100 mm [17, 18]. This method allows for a more fine-grained rating of musculoskeletal discomfort than e.g. a 10-point visual analogue scale. Any discomfort reported immediately before a standing session was subtracted from subsequently reported discomfort for each body part, with a minimal possible value of 0 mm. For analysis purposes, total discomfort ratings at time points 0, 60, 120, 180 for all three sessions and 240 min in the standing session were presented and statistically analyzed.

##### Leg swelling

Immediately pre- and post-each session, ankle girth of both sides was recorded using a soft tape measure to get an estimate of lower extremity edema. An absolute pre- and post-condition difference of the mean value of left and right ankle girth was reported.

#### Primary outcome measure: oxygen consumption (VO_2_)

Oxygen consumption of the participants was measured by indirect calorimetry using the Cosmed K4b2 gas analyzer (Cosmed, Rome, Italy). For the sitting condition, a final oxygen uptake was calculated as a mean of four 10-min measurements taken at time points 1–10 min, 50–60 min, 110–120 min, and 170–180 min. For both static and dynamic standing conditions an additional measurement was taken into consideration at time point 230–240 min.

#### Secondary outcome measure: *Cognitive performance*

Two sets of cognitive tests were assessed during sitting and both standing sessions. First, the Trail Making Test (TMT) from the Delis-Kaplan Executive Function System (D-KEFS) was used to assess set shifting and executive functioning. All five conditions were assessed and the total time taken to complete each is presented. TMT-1 measures visual scanning, TMT-2 number sequencing, TMT-3 letter sequencing, TMT-4 number-letter switching, and TMT-5 motor speed [19]. Second, an analog version of the Stroop test (Stroop Color-Word test) was also used [20]. Stroop-1 measures word naming/reading, Stroop-2 measures color naming, Stroop-3 measures verbal response inhibition, and Stroop-4 measures inhibition/switching. The time taken was recorded in seconds for each Stroop condition.

### Statistical analysis

Data were analyzed in SPSS software version 25.0 (IBM, Armonk NY, USA). Parametric data were entered into a 2 × 2 mixed design repeated measures analyses of variance (ANOVA) with conditions Sitting, Static standing and Dynamic standing as the within subject variable, with post hoc comparisons carried out with the Bonferroni correction method for multiple testing. Non-parametric data were analyzed with Friedman’s ANOVA. Significant results were further evaluated in post hoc pairwise comparisons using a Wilcoxon Signed-Ranks Matched-Pairs test. Best fit line plotting was performed to determine the rate of total musculoskeletal discomfort development. An alpha below 0.05 indicated statistical significance.

## Results

### Feasibility of standing in older adult diabetics

There were no screen failures and no study drop-outs. All participants that were initially screened and included in the study completed all three sessions without any missing data. Sitting (baseline) sessions lasted on average 180.5 ± 0.8 min, while static and dynamic were on average 240.5 ± 0.9 and 240.4 ± 0.7 min long, respectively.

#### Primary outcome measure: oxygen consumption

A significant effect of condition was found for VO_2_ consumption (F_2,22_ = 40.862, *p* < 0.001, partial η^2^ = 0.788). In comparison to sitting, VO_2_ significantly increased by 28.0% (*p* < 0.001) and 34.5% (*p* < 0.001) during static and dynamic standing, respectively. Furthermore, there was a non-significant trend between both standing conditions, where dynamic standing was found to be 5.3% (*p* = 0.052) higher VO_2_ intake (see Table 3).

#### Secondary outcome measures: net standing duration, rate of musculoskeletal discomfort development, leg swelling and cognition

Table 2 provides the summary descriptive statistics of user behavior and experience of table use.

**Table 2:** Results of descriptive measures of user behavior and experience of table use (average ± standard deviation)

	Sitting	Static standing	Dynamic standing
Number of breaks (N)	0.7 ± 0.8	2.3 ± 2.2*	2.0 ± 2.2*
Duration of breaks (min)	1.9 ± 2.4	17.3 ± 29.3*	15.3 ± 28.5*^#^
Overall movement (counts/min)	26.3 ± 12.5	43.7 ± 29.4^$^	60.4 ± 31.8*^#^
Rate of total musculoskeletal discomfort development (mm/min)	0.02 ± 0.04	0.39 ± 0.53*	0.23 ± 0.37*^#^
Average swelling for both legs (cm)	0.11 ± 0.17	0.64 ± 0.61*	0.44 ± 0.48^$^

*Note:* Main effects are reported in the result section, however, post hoc tests are represented as follows: different from Sitting: *p < 0.05 (^$^ trend < 0.10); different from Static standing: ^#^*p* < 0.05 (^†^ trend < 0.10)

### Number and duration of breaks taken during sitting and standing conditions

As expected, patients took more breaks during both standing conditions as compared to sitting (χ^2^(2) = 13.923, *p* = 0.001). Post hoc testing revealed a significant increase in the number of break periods from sitting (median = 1 break) to static standing (*Z* = -2.536, *p* = 0.011) as well as from sitting to dynamic standing (*Z* = -2.410, *p* = 0.016), while static (median = 3 breaks) and dynamic (median = 2 breaks) standing did not differ (*Z* = -1.633, *p* = 0.102) in the number of breaks taken. Breaks during sitting represented mainly restroom use while breaks during the standing conditions represented both restroom breaks as well as short duration of sitting. The total duration (in minutes) spent on breaks significantly differed between the three conditions (χ^2^(2) = 13.027, *p* = 0.001). Post hoc test revealed a significant increase of duration of breaks from sitting (median = 3 min) to static (median = 8 min) standing (*Z* = -2.807, *p* = 0.005) as well as from sitting to dynamic (median = 6 min) standing condition (*Z* = -2.320, *p* = 0.020). Duration of break periods was longer during static as compared to dynamic standing sessions (*Z* = -2.252, *p* = 0.024).

### Total movements

Overall movements, measured as total activity counts, progressively increased from sitting to static and dynamic standing (F_2,22_ = 40.862, *p* = 0.001, partial η^2^ = 0.461). In comparison to sitting, total amount of activity counts also significantly increased by 182.2% (*p* = 0.003) during dynamic standing. When comparing sitting to static standing, there was a near-significant increase of 113.7% of total movement (*p* = 0.056). Dynamic standing activity counts increased by 65% compared to the static standing session (*p* = 0.024).

### Musculoskeletal discomfort rates

**Fig. 1:**
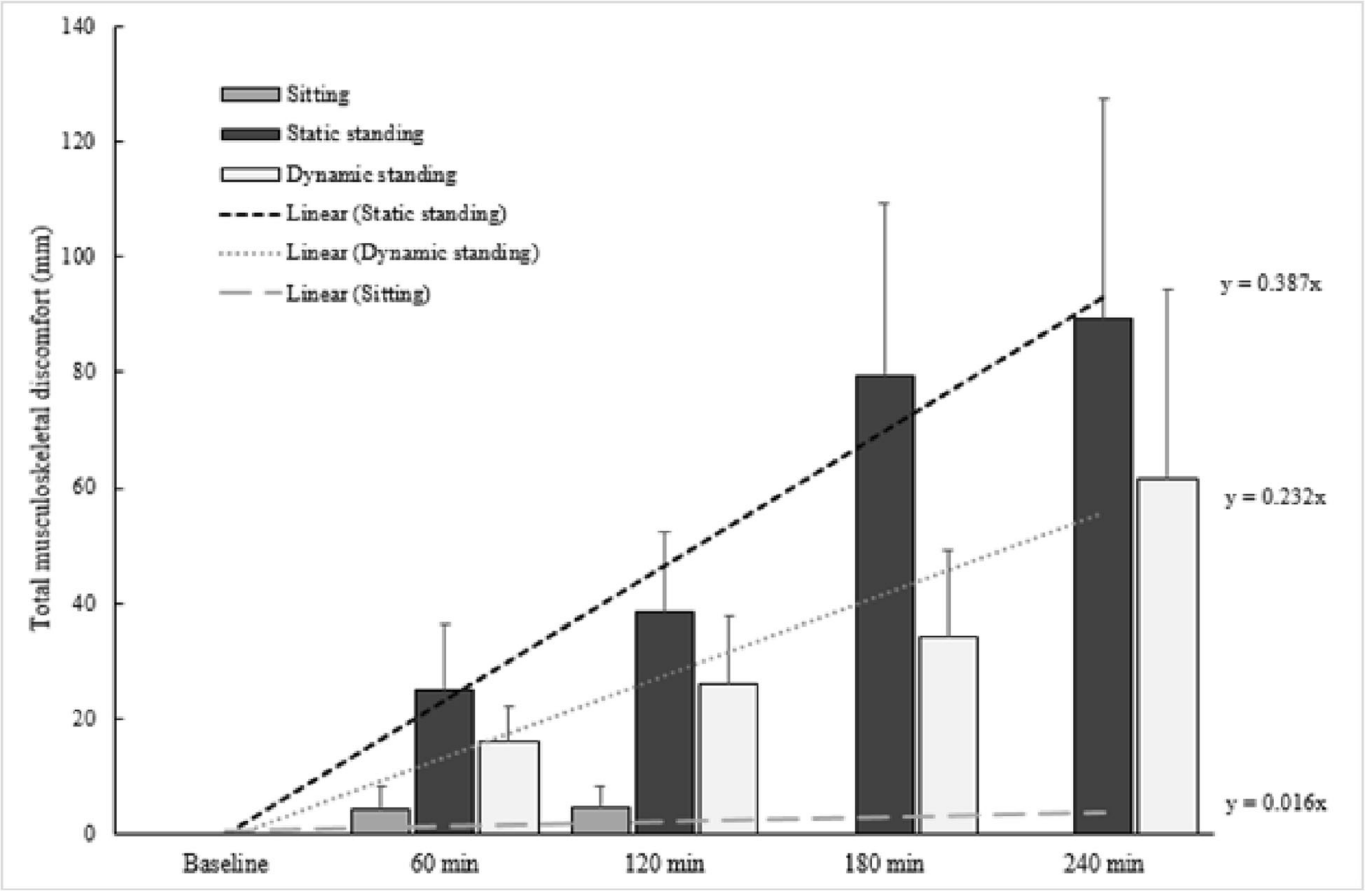
Total and rate of musculoskeletal discomfort during 3h of sitting as well as 4h of static and dynamic standing. *Note:* Group means (±1 standard error of the mean) are presented for all three conditions. For the Sitting condition all participants reported 0 musculoskeletal discomfort at the 180-min point

Figure 1 depicts average discomfort ratings over time for each of the sessions (sitting, static and dynamic standing), as well as their linear approximations of discomfort development rate.

The rate of total musculoskeletal discomfort development (mm/min) significantly differed between the three conditions (χ^2^(2) = 10.889, *p* = 0.004), with significant increases from sitting to static standing (*Z* = -2.201, *p* = 0.028) and from sitting to dynamic standing (*Z* = -2.366, *p* = 0.018). Finally, the dynamic standing condition (*Z* = -2.028, *p* = 0.043) showed a lower rate of total musculoskeletal discomfort development when compared to the static standing condition.

### Leg swelling

Overall leg swelling was low (less than 1 cm for all conditions), however, it significantly differed between the three conditions (χ^2^(2) = 7.588, *p* = 0.023). Compared to sitting, leg swelling was significantly higher for static standing (*Z* = -2.539, *p* = 0.011) and non-significantly higher for dynamic standing (*Z* = -1.758, *p* = 0.074). There was no difference in leg swelling between static and dynamic standing (*Z* = -1.122, *p* = 0.262).

#### Cognitive performance

Table 3 lists cognitive outcome measures during each session.

**Table 3:** Primary and secondary outcome measures of sitting as well as static and dynamic standing sessions (average ± standard deviation)

	Sitting	Static standing	Dynamic standing	*P*_condition_ (partial η2)	Δ _Static - Sitting_	Δ _Dynamic - Sitting_
Primary outcome:						
Oxygen uptake VO_2_ (ml/min/kg)	3.3 ± 0.6	4.2 ± 0.6*	4.4 ± 0.6*^†^	< 0.001 (0.788)	0.87 ± 0.47	1.08 ± 0.49^¥^
Secondary outcome						
TMT1: Visual Scanning (sec)	26.8 ± 9.9	23.3 ± 3.8	24.6 ± 7.4	0.228	−3.47 ± 8.18	−2.19 ± 7.45
TMT2: Number Sequencing (sec)	43.6 ± 10.9	32.9 ± 11.7*	37.0 ± 17.9	0.022 (0.293)	−10.71 ± 11.58	−6.53 ± 14.42
TMT3: Letter Sequencing (sec)	42.6 ± 13.2	34.9 ± 9.2	36.1 ± 15.5	0.055	−7.72 ± 11.39	−6.55 ± 11.25
TMT4: Number-Letter Switching (sec)	93.0 ± 43.5	80.7 ± 53.0	90.6 ± 53.3	0.221	−12.28 ± 17.54	−2.41 ± 27.58
TMT5: Motor Speed (sec)	36.9 ± 11.0	33.9 ± 14.0	31.8 ± 12.7*	0.022 (0.292)	−2.93 ± 6.37	−5.09 ± 6.38
Stroop1: Word naming/reading (sec)	54.8 ± 7.3	51.6 ± 7.9*	52.3 ± 8.8^$^	0.032 (0.269)	−3.17 ± 4.02	−2.42 ± 4.08
Stroop2: Color naming (sec)	72.3 ± 16.9	67.4 ± 17.1*	68.0 ± 15.8*	0.018 (0.306)	−4.83 ± 6.51	−4.25 ± 4.90
Stroop3: Verbal response inhibition (sec)	123.2 ± 28.6	109.8 ± 23.8*	114.2 ± 33.6^$^	0.013 (0.328)	−13.42 ± 9.55	−9.00 ± 16.83
Stroop4: Inhibition/switching (sec)	142.6 ± 30.8	123.1 ± 25.6*	125.1 ± 35.5*	0.001 (0.457)	−19.50 ± 12.60	−17.50 ± 20.98

*Note:* TMT Trail Making Test; Main effects are reported in the results section, however, post hoc tests are represented as follows: different from Sitting: *p < 0.05 (^$^ trend < 0.10); different from Static standing: ^#^p < 0.05 (^†^ trend < 0.10)

There were no negative effects for cognitive measures of set shifting (TMT) or response inhibition (Stroop test) during the standing sessions compared to sitting. Exploratory analyses showed limited but significant beneficial effects of session condition for all Stroop sub-sets as well as TMT sub-tests TMT2 and TMT5 (Table 3).

## Discussion

Our findings show that ad libitum four-hour standing is feasible and may have clinical benefits in an older adult type 2 diabetic population. Compared to sitting, oxygen consumption increased during static standing with an additional trend for higher energy expenditure during dynamic standing. These results suggest that standing, as a rudimentary form of non-exercise physical activity, should be further explored as a simple yet effective means to counteract sedentary behavior in this patient population. Moreover, making small weight-shifting movements during standing may be even more beneficial than regular stance. For example, our results also showed that shorter breaks were needed during dynamic compared to static standing. The rate of total musculoskeletal discomfort development was also lower in the dynamic compared to the static standing group.

Increased sedentary behavior in adults is not only associated with T2DM but also with other deleterious health outcomes, such as cancer, cardiovascular disease incidence and increased overall mortality [21]. Changing sedentary behavior to a more physically active lifestyle in older adults remains challenging [22]. For example, despite well-known physical exercise-related health benefits, in general, the older adult population remains sedentary for an average of 9.4-h a day (up to 80% of their waking day) [23]. Diabetic patients engage even 10–20% less frequently in physical activities [24–26]. These observations indicate a clinically unmet need to promote physical activity in older adult patient populations who need it the most. Given the well-known barriers to exercise participation in older adult patient populations with chronic disorders, a natural first step would be to reduce sedentariness with intermittent non-exercise physical activity. To our knowledge, this is the first study to investigate the feasibility and clinical effects of prolonged desktop standing in rather inactive and overweight patients with T2DM, and therefore may hold clinical and public health relevance.

Energy expenditure of physical activity other than volitional exercise is referred to as non-exercise activity thermogenesis (NEAT) [27]. Previous research found that NEAT is lower in obese people [28]. Standing may be a simple, yet effective, means to increase NEAT and counteract the negative effects of physical inactivity due to prolonged inactivity [29]. Our results are in line with previous studies showing elevated VO_2_ consumption rates during (both) standing condition(s) as compared to sitting [30]. Interestingly, compared to the study by Cox et al. we had similar VO_2_ values for the seated condition (3.3 ml·kg^− 1^·min^− 1^), however, our T2DM participants showed higher mean values of VO_2_ consumption (4.2 ml·kg^− 1^·min^− 1^) during regular stance compared to lean the lean and asymptomatic participants in their study (3.6 ml·kg^− 1^·min^− 1^) [30]. This observation suggests that standing may potentially have even more benefits in an overweight population of older T2DM patients. We also found a trend toward additional higher energy expenditure in our T2DM patients during dynamic standing compared to regular or static standing. As such, use of a dynamic standing desk may be of particular relevance for older overweight adults with T2DM as it increases NEAT without significant side-effects, such as significantly lower discomfort rates [31].

There were no negative effects of standing on cognitive functions, such as executive functions of set shifting and response inhibition as measured by the Trail Making and Stroop tasks, respectively. Most of prior studies on sit-stand, bike and treadmill desks were aimed at reducing sedentary behavior in the work place, especially among office workers. In office workers, it is crucial that such physical activity interventions do not negatively impact a person’s productivity, their efficiency of daily tasks, or general cognitive performance. However, results of these studies showed relatively mixed results with respect to the outcome of these physical activity intervention on cognitive-motor performance or work productivity. For example, Koren et al. [32] introduced a cycloergometer to office workers with the aim of achieving minimum standards for daily physical activity during working hours. These authors found evidence of increased typing time but without other negative cognitive outcomes. Treadmill desk studies revealed a significant reduction in sedentary behavior during working hours [33] but with relatively mixed results in cognitive, fine motor skills and work performance (e.g. [34–37]). Even though treadmill desks may provide particular benefit to overweight and obese individuals, two recent systematic reviews reported possible detrimental effects on work productivity as well as motor abilities [38, 39]. In contrast, our findings do not show evidence of any detrimental cognitive executive function effects during static or dynamic desktop standing session in our older adult T2DM patient population. Further research is needed to determine whether dynamic types of desktop standing may provide more safe alternatives to increase physical activity in office workers without adverse effects on job productivity.

A limitation of our study was that we did not specifically assess metabolic glycemic measures in our patients. There is existing evidence that simple standing may have beneficial metabolic effects in diabetic populations. For instance, Henson et al. [29] reported reduced postprandial glucose, insulin, and nonesterified fatty acids after 5-min bouts of standing or light walking in postmenopausal women at high risk of T2DM. In a similar attempt in a population of 24 obese older adults with T2DM, Dempsey et al. [40] found attenuated postprandial glucose, insulin, C-peptide, and triglyceride levels after 3-min bouts of light-intensity walking and simple resistance activities. Both studies concluded that these simple behavioral approaches might represent a novel public health intervention aiming at reducing the risk of T2DM and/or improving metabolic profile of such symptomatic individuals [29, 40]. Future clinical trials should be conducted to examine whether our desktop standing interventions may have similar benefits in a population with T2DM, while taking into consideration that dynamic standing is, according to relative VO_2_ calculations, approximately two times less energetically demanding as light-intensity walking [40].

An important finding of our study is that prolonged (four-hour) static and dynamic standing is feasible and safe in a rather sedentary and overweight older adult patient population with T2DM. However, three main methodological limitations should be taken into consideration when interpreting the results of the present study. First, the laboratory setting might have enticed our participants to accomplish all requested conditions; therefore, testing in a home environment is necessary to reassess our paradigm and its long-term utilization. Second, patients were randomized to the standing sessions after completion of a sitting session. Therefore, learning effects may affect cognitive outcome measures and could potentially explain the trends toward improved cognitive performance during the standing sessions in our study. However, participants received detailed instructions as well as a test trial in order to mitigate the learning effect prior to starting the baseline (sitting) condition. Other factors such as exercise-level associated arousal may also influence participants’ cognitive performance [41–43]. Third, the number of subjects was small in our feasibility testing resulting in lack of statistical power. Future phase 2 clinical trials in a larger population are needed to address these important issues. These studies should also include specialized laboratory test (blood glucose, insulin resistance/sensitivity etc.) and further assess metabolic dose-response effects of standing duration taking into consideration also individual baseline physical activity levels. While evaluating the effects of non-exercise regimes (e.g. dynamic standing) on cognitive functioning and work productivity, transition states and thus multiple time-point testing of cognitive abilities should be considered [44].

## Conclusion

In conclusion, our study indicates that patients with T2DM are able to perform longer duration standing sessions without any serious side effects. Four hour standing sessions are therefore feasible in older adult rather sedentary and overweight patients with T2DM and, more importantly, can possibly have metabolic benefits on energy expenditure. Standing interventions in patients with T2DM, especially when combined with weight-shifting movements, can represent a novel, barrier-free non-exercise physical activity program, that targets sedentariness and can be easily and safely integrated into daily routines. Further research is needed to assess long-term effects of such standing interventions in the home environment in older adult patients with T2DM.

## Data Availability

Data are available from the corresponding author upon reasonable request.

## Abbreviations

T2DM: Type 2 Diabetes Mellitus
NEAT: Non-exercise activity thermogenesis
OSHA: Occupational Safety and Health Administration
VO2: Oxygen consumption
D-KEFS™: The Delis-Kaplan Executive Function System™
TMT: Trail Making Test
ANOVA: Analysis of Variance
